# Supplementary material for: A cross-sectional evaluation of five warfarin anticoagulation services in Uganda and South Africa
Source: PLoS One. 2020 Jan 29;15(1):e0227458. doi: 10.1371/journal.pone.0227458 (PMC6988943; doi:10.1371/journal.pone.0227458)
Supplement: S1 Appendix — (PDF) [file pone.0227458.s001.pdf]

# APPENDIX: DATA COLLECTION FORM

Clinic Name ____________________________ Date of Assessment __ / __ / __

## Clinic Service Provision

Level of service provision — Primary ☐ Secondary ☐ Tertiary ☐

Cadre of clinician providing care — Specialist ☐ Nurse ☐ Medical officer ☐ Clinical officer ☐

Information provided to patient — Counselling ☐ Information sheets ☐ Other ______________

Number of clinics per week ___________ Number of patients seen in past 4 weeks___________

Is point of care INR testing provided — Y ☐ N ☐

Average waiting time for INR results — Same day ☐ 1 day ☐ 2 days ☐ Other ______________

Is there a fee to attend clinic — Y ☐ N ☐ If Y, specify _________

Does the patient pay for INR testing — Y ☐ N ☐ If Y, specify _________

Describe the follow-up schedule: Weekly ☐ Monthly ☐ 3-monthly ☐ 6-monthly ☐ Other_______

What is the response to out of range INR? — Increase clinic frequency ☐ Counselling ☐ Dose adjustment ☐ Other ___________________

How are defaulters managed? ________________________________________________________________

## Drug prescribing and availability

What is the relationship with the pharmacy? On-site, part of clinic ☐ On site, near the clinic ☐
Separate ☐

Are standard guidelines for the initiation of warfarin available? Y ☐ N ☐ If Y, specify_________

What is the target INR range for patients with: AF _______ DVT ________ PE ________ Heart Valve Disorders ________

What is the standard warfarin loading regimen, and is this same for all indications of anticoagulation?

_____________________________________________________________________________________

Do any individual patient factors alter the loading dose? Y ☐ N ☐

If Y, which: Age ☐ Weight ☐ Comorbidities ☐ Concomitant meds ☐

Is warfarin dispensed to patients at the same facility? Y ☐ N ☐

Are the following alternatives available? Dabigatran ☐ Rivaroxaban ☐ Apixaban ☐ Other_______

Do patients pay for medication? Y ☐ N ☐ If Y, specify_________

Is aspirin substituted or provided instead of warfarin? E.g for AF Y ☐ N ☐

Is heparin used for the initial treatment of PE/DVT? Y ☐ N ☐

## Guidance available for warfarin-dose adjustment

Is there an SOP/protocol/protocols for INR-guided dose adjustment of warfarin? Y ☐ N ☐

If Y, specify which ________________________ Is it referenced? ☐ Is it validated for local population? ☐

When dosing is adjusted what is the average frequency of subsequent INR checks? _______________________

In the event of a high INR how soon is an INR repeated? ______________________________________________

Would vitamin K be administered to participants with an INR over 8? Y ☐ N ☐

## Folder and electronic record review

Age ____ years Sex M☐ F☐

Indication for anticoagulation VHD☐ AF☐ DVT☐ PE☐ Other (specify) ___________________

INR target 2-3 ☐ 2.5-3.5 ☐ 3-4 ☐ Other (specify) ___________________

Comorbidities: TB ☐ HIV ☐ RHD ☐ Others (specify)__________________

Date of warfarin initiation __ / __ / __

Concomitant medication

| Drug | Dose | Date of initiation | Indication |
|---|---|---|---|
| ___________ | __________ | __ / __ / __ | ______________________________________ |
| ___________ | __________ | __ / __ / __ | ______________________________________ |
| ___________ | __________ | __ / __ / __ | ______________________________________ |

Number of clinic visits over last 6 months 1 ☐ 2 ☐ 3 ☐ 4 ☐ 5 ☐ 6 ☐ Other_____

INR results over last 6 months:

| INR | Date | INR | Date | INR | Date |
|---|---|---|---|---|---|
| ______ | __ / __ / ___ | ______ | __ / __ / ___ | ______ | __ / __ / ___ |
| ______ | __ / __ / ___ | ______ | __ / __ / ___ | ______ | __ / __ / ___ |
| ______ | __ / __ / ___ | ______ | __ / __ / ___ | ______ | __ / __ / ___ |
