# Supplementary material for: A cross-sectional evaluation of five warfarin anticoagulation services in Uganda and South Africa
Source: PLoS One. 2020 Jan 29;15(1):e0227458. doi: 10.1371/journal.pone.0227458 (PMC6988943; doi:10.1371/journal.pone.0227458)
Supplement: Attachment — Submitted filename: Reviewer PlosOne.docx [file pone.0227458.s003.docx]

**Reviewer # comments**

Manuscript title: A cross-sectional evaluation of warfarin anticoagulation services in Uganda and South Africa

General feedback: The paper addresses an important topic on warfarin in the lower-income country (Uganda) and middle-income country (South Africa)

However, several methodological issues need to be addressed.

1. Methods:
   a. The audit was conducted between 1 January and 31 July 2018. Authors extracted demographic data and clinical data including indication for anticoagulation, comorbidities, concomitant medications and INR results. It would have been essential to state the span over which INR results were collected. Did the authors document all the INR results over the study period (1 January and 31 July 2018)?
   b. *We selected, through convenience sampling, key members of clinic staff who were involved in anticoagulation care and who indicated their availability and willingness to be interviewed.* *Interviews were conducted using a structured questionnaire*. I would avoid the word interview but rather use questionnaire administration. This was not a qualitative study, but rather the administration of structured questionnaires.
2. Statistical and data analysis
   a. Although the authors stated that time in therapeutic range (TTR) over the most recent

three months was calculated using the Rosendaal interpolation method, one would expect more description as to who qualified for analysis. The Rosendaal method uses INR values from patients with at least two valid intervals separated by 56 days (8 weeks) or less, without an intervening hospitalisation. Some authors have used 60-90 days between readings. It is, therefore, essential to state the interval.

3. Results:
   a. A lot of the initial sentences in the results section fits more on the method section than as results. For instance, the description of the study setting. Knowing that Mulago is a tertiary hospital or Groote Schuur as a secondary hospital is a piece of basic information that describes a study site, even before data collection. Kindly review this part.
   b. Kindly state if valvular heart diseases included mechanical heart valves.
4. Discussion
   a. Although the authors pointed out that the cost of INR testing and warfarin is may be prohibitive in anticoagulation control, this was not clear in figure 1. Ugandan site where patients paid for the warfarin/INR service had a similar level of anticoagulation control as the South African site! I expected this observation to feature in the discussion than the way the authors discussed.
   b. Similarly, the contribution of HIV was not very evident despite the considerable difference in the prevalence (20% vs 5%). As in (a) above, this was not clearly stated.
   c. Although this study is typically descriptive and may not be powered enough to analyse associations, it would have been nice to look at whether the variables which were collected had any influence on the level of anticoagulation control. If this is not done, the discussion on ‘’associated factors’’ will be limited.
   d. Study limitations. How about the sample size? Was it enough to answer the primary objective? This is not justified in the manuscript and could feature as a limitation.
   e. Conclusion: *The findings from our work will inform further research to develop and validate a warfarin dosing algorithm for use in this setting. Was there any evidence to suggest that the absence of a validated algorithm affected anticoagulation control?* As some sites had adopted some algorithm, one would expect the authors to have analysed if the lack of an algorithm affected anticoagulation control before having this conclusion.
