# Supplementary material for: A cross-sectional evaluation of five warfarin anticoagulation services in Uganda and South Africa
Source: PLoS One. 2020 Jan 29;15(1):e0227458. doi: 10.1371/journal.pone.0227458 (PMC6988943; doi:10.1371/journal.pone.0227458)
Supplement: Attachment — Submitted filename: Response to reviewers.docx [file pone.0227458.s004.docx]

20^th^ November 2019

Joel Msafiri Francis, MD, MS, PhD

Academic Editor

PLOS ONE

Dear Dr Francis,

Many thanks for the comments on the manuscript titled, “A cross-sectional evaluation of warfarin anticoagulation services in Uganda and South Africa”.

Further to these comments, please see below responses to the points raised and attached revised manuscript.

| **Reviewer #1 Comment** | **Response** |
| --- | --- |
| **Title:** Consider clarifying the title to avoid misunderstanding that the evaluation was performed on a nationwide scale (e.g. “A cross-sectional evaluation of warfarin anticoagulation services in five clinics in Uganda and South Africa”). | We have changed the title to “A cross-sectional evaluation of five warfarin anticoagulation services in Uganda and South Africa” in response to this comment |
| **Abstract**: Page 3 (Line 55): Consider using more specific language than “INR control”. This is acceptable clinical wording, but the conclusion suggested by this manuscript specifies inadequate TTR attainment is the finding, which may be a more precise description of the reported outcome | We have replaced “INR control” with “TTR” in the conclusion of the abstract, and in the discussion of the manuscript, lines 170 and 216. |
| **Introduction:** - Page 4 (Line 68): Citation needed. - Page 4 (Line 70): Citation needed | We have added the following citations:  WHO. Global tuberculosis report 2019. 2019 ISBN 978-92-4-156571-4  C Sekaggya, D Nalwanga, A Von Braun, R Nakijoba, A Kambugu, J Fehr ML and BC. Challenges in achieving a target international normalized ratio for deep vein thrombosis among HIV-infected patients with tuberculosis: a case series. BMC Hematol [Internet]. 2016;16(1):1–5. Available from: http://dx.doi.org/10.1186/s12878-016-0056-6 |
| **Methods**: - Page 5 (Line 81): Was there a reason that a 7 month data collection period was selected? It seems that 12 months would be a more relevant/representative timeframe, particularly since there are seasonal variations in hospitalization rates and causes, which may impact the generalizability of the findings. If there was not a specific reason for the selected time interval, this should be reported as a limitation of the study. | This study focuses on outpatients in chronic care rather than exploring rates and causes of hospitalisation in acutely presenting patients. It is true that there may be some seasonal variation in number of patients seen at a clinic, but the patients whose records were audited are in chronic care.  We did not continue to collect data over the full 7 months at any one facility. All included clinics were audited within the 7-month time window that we report.  In response to this comment we have added the following to the limitations in the discussion: “We audited each included facility at a single timepoint.” |
| Page 5 (Line 93): Please describe the rationale and specific procedure for the convenience sample. Were patients enrolled consecutively? Were they enrolled following a clinic visit? Was there any random selection procedure of charts? Enrolment alphabetically could potentially introduce complicating factors such as overrepresentation of certain families, ethnic groups, etc. | We didn’t enrol patients for the audit. There was no direct interaction with patients. Clinical information was collected by retrospective record review of clinical records, as is stated in the methods.  The method of sampling clinical records review varied between clinic sites because of different systems for record storage and documentation of clinical attendance between clinics. We aimed to minimise bias but did not have a sampling frame from which to draw a truly random sample at any of the included clinics.  At Guguletu CHC in Cape Town we used a random number sequence to select a sample of patient records from the four filing cabinets in which they were stored. At Groote Schuur (which runs 4 clinics a week), we used a systematic sampling method: We calculated “n” for each day based on number of patients who visited the clinic that day, so that we would review 5 records per clinic. We generated a random number between 1 and 8 in Excel to flag the first record to review, then reviewed every nth record thereafter. At Tygerberg we took a convenience sample- we visited each clinic once, and included clinical records of all patients that were seen at that clinic on that day.  At Mulago National Referral Hospital, we sampled every other file from the total number of records of patients on warfarin  At Uganda Heart Institute where patients are reviewed on weekdays, we took a convenience sample from records of patients that had not come for review on the days of data collection  In response to this comment we have added the following to the methods:  “The method of sampling clinic records varied between clinic sites because of difference in systems for record storage and documentation of clinic attendance. We aimed to minimise bias but did not have a sampling frame from which to draw a truly random sample at any of the included clinics.”  We also deleted the word “convenience” from the methods section of the abstract and the manuscript. |
| Page 5 (Line 94): If a hypothesis of this paper is to assess for potential medication interactions between warfarin and TB/HIV drugs, perhaps it would make more sense to select and query the percentage of patients taking medications known a priori to affect warfarin metabolism. Examples of such medications (NNRTIs and rifampicin) are brought up by the authors in the Discussion section (Page 11) but are not reported as included among drugs queried in the study design | We extracted data on all concomitant medications from patients’ clinic records, as is stated in the 3^rd^ paragraph of the methods (pg 5)  We used “Stockley’s Drug interactions” to determine which of the concomitant medications had a potential drug interaction with warfarin. This has been added to the methods under “Data analysis”.  In response to this comment we have added the following to the text (last paragraph of results, p 9:  “We found that 126 patients (55%) had at least one concomitant medication which could potentially interact with warfarin [10]. There were 12 patients (5.2%) on efavirenz and one patient (0.43%) on lopinavir-ritonavir.” |
| Page 5 (Line 96): “Statistical and data analysis” is redundant. The subheading can be changed to “Statistical Analysis” or “Data Analysis”. | We have changed this sub-heading to “Data Analysis”. |
| Page 5 (Line 98): Please specify which summary statistics were used (e.g. median, IQR, etc.). | We have specified the summary statistics used |
| Page 5 (Line 99): Please name what software packages were used for statistical analysis and figure generation. | We used Stata 15.1 (StataCorp, College Station, Texas, USA) and R software package 3.5.3 for statistical analysis and figure generation. This has been added to the methods. |
| Page 5 (Line 99): This study could be strengthened by performing regression analyses, which could potentially identify factors that may be associated with substandard TTR attainment. Was there a reason this was unable to be performed? | The current study was purely an audit of current anticoagulation practice, to provide insight into current practices and level of control in the study settings. Our intention was not to attempt to identify factors associated with INR control, and as such our study was not powered to do so.  We have undertaken a further, adequately powered prospective study to investigate factors associated with warfarin therapeutic dose and INR control- this study is currently recruiting participants. |
| Page 6 (Line 104): Please include the basis for waiving of informed consent from patients | We did not directly interact with patients for purposes of this audit. This study was a retrospective review of routinely collected clinical data. Neither ethics committee require obtaining informed consent from patients for retrospective clinical record reviews. |
| Page 6 (Line 109): Please include the basis for waiving of informed consent from staff | In South Africa, we had to approach multiple staff members at the participating healthcare facilities in order to collect the required information. Since the information we were collecting did not include any questions of a sensitive or personal nature, the UCT Human Research Ethics Committee waived requirement for obtaining informed consent from staff members from whom we collected information. We only interviewed staff after obtaining permission from the participating health care institutions. |
| **Results**: - Page 6 (Lines 111-117): The descriptions of the audited clinics and interviewed individuals could be included in the Study Design and Sites subsection rather than the Results section, since these were known prior to the acquisition of study data. | In response to this comment we have moved the description of the sites to the Methods section. |
| Page 8 (Line 133, Line 136): Please clarify the analyses were performed in “Ugandan sites” and “South African sites” rather than “Uganda” or “South Africa”. | We have revised the text- it now reads “Ugandan clinics” and “South African clinics”. |
| Discussion: - Page 10 (Line 166): Please clarify how regular patient follow-up and regular INR monitoring were confirmed in this present analysis (e.g. what percentage of patients in the program actually made it to monthly follow-up appointments or INR monitoring visits). | We collected information on INR monitoring as part of this audit. The median number of INR overall was 4 in 6 months (IQR 3 to 6); 3 (IQR 3 to 4) at Ugandan clinics and 5(IQR 3 to 6) at South African clinics- see table 2.  We didn’t collect information on dates of clinic visits.  We have revised the sentence to read “This occurred despite ~~regular patient follow-up and~~ regular INR monitoring”. |
| Page 11 (Lines 189-193): See above in Methods section. The authors discuss the potential of NNRTIs or rifampicin to affect warfarin metabolism, but the prevalence of use of these drugs is not explicitly queried in the study design. | We collected information on all concomitant medication, as many medicines are known to potentially interact with warfarin. This is stated in the methods (third paragraph under heading “Study design and sites” p 5). This included collecting information on concomitant antiretroviral and antituberculosis drugs.  We have expanded table 2 to include the 10 most commonly prescribed concomitant medicines, which includes efavirenz at the Ugandan clinics |
| **Reviewer #2 Comment** | **Response** |
| 1. Authors state that they used a convenience sample. What was this convenience? Was it consecutive patients seen in the clinics? Random? Needs some elaboration. | See response to reviewer one above- we have expanded the description of sampling, and deleted the word “convenience” in response to both reviewers’ comments. |
| 2. The authors stated that they interviewed service providers (Doctors, nurses, etc.). However, data obtained from these interviews (quantitative or qualitative) are not clearly mentioned or discussed in the results or discussion sections. Most of the data they state in results section could have come from the patients and patient records. This needs to be clarified | We have included the data capture sheet used to collect information from the clinic staff and the data capture sheet for the clinical record review in the supplementary material.  We did not collect any information directly from patients. |
| **Reviewer #3 Comment** | **Response** |
| a. The audit was conducted between 1 January and 31 July 2018. Authors extracted demographic data and clinical data including indication for anticoagulation, comorbidities, concomitant medications and INR results. It would have been essential to state the span over which INR results were collected. Did the authors document all the INR results over the study period (1 January and 31 July 2018)? b. We selected, through convenience sampling, key members of clinic staff who were involved in anticoagulation care and who indicated their availability and willingness to be interviewed. Interviews were conducted using a structured questionnaire. I would avoid the word interview but rather use questionnaire administration. This was not a qualitative study, but rather the administration of structured questionnaires. | INR results were collected over the six months prior to the most recent clinic visit. This has been clarified in the methods.  We used a structured data capture sheet to collect information. In response to this comment we have added the data capture sheets as supplementary material  and we have revised the sentence describing data collection from clinic staff to read as follows:  “We ~~interviewed~~ collected information from key members of clinic staff, including doctors, nurses, laboratory and pharmacy staff, using a structured ~~questionnaire~~ data capture sheet […] (see data capture tool in the supplementary material).”  The data capture sheet was not a formal questionnaire- we have therefore not used the term “questionnaire”. |
| 2. Statistical and data analysis a. Although the authors stated that time in therapeutic range (TTR) over the most recent three months was calculated using the Rosendaal interpolation method, one would expect more description as to who qualified for analysis. The Rosendaal method uses INR values from patients with at least two valid intervals separated by 56 days (8 weeks) or less, without an intervening hospitalisation. Some authors have used 60-90 days between readings. It is, therefore, essential to state the interval. | We did not place any restrictions on the maximum allowed time interval between consecutive INR measurements in the TTR calculation.  In response to this comment, this has been clarified in the methods by addition of the following:  “We did not place any restriction on the maximum interval between INR measurements when applying the method.” |
| Results:   a. A lot of the initial sentences in the results section fits more on the method section than as results. For instance, the description of the study setting. Knowing that Mulago is a tertiary hospital or Groote Schuur as a secondary hospital is a piece of basic information that describes a study site, even before data collection. Kindly review this part b. Kindly state if valvular heart diseases included mechanical heart valves. | In response to this comment and the comment of reviewer 1, we have moved the description of sites to the Methods section  Valvular heart diseases does include mechanical heart valves.  We have added this to the results in response to this comment. The sentence now reads: “The three most common indications for warfarin treatment were venous thrombo-embolism (49%), atrial fibrillation (32%) and valvular heart disease (including mechanical heart valves) (13%).” |
| Discussion:   a. Although the authors pointed out that the cost of INR testing and warfarin is may be prohibitive in anticoagulation control, this was not clear in figure 1. Ugandan site where patients paid for the warfarin/INR service had a similar level of anticoagulation control as the South African site! I expected this observation to feature in the discussion than the way the authors discussed b. Similarly, the contribution of HIV was not very evident despite the considerable difference in the prevalence (20% vs 5%). As in (a) above, this was not clearly stated. c. Although this study is typically descriptive and may not be powered enough to analyse associations, it would have been nice to look at whether the variables which were collected had any influence on the level of anticoagulation control. If this is not done, the discussion on ‘’associated factors’’ will be limited. | This study was purely an audit of current anticoagulation services, to provide insight into current practices and INR control in the study settings. The study was not powered to identify associations with poor INR control  There are many potential contributing factors to poor INR control. These include drug costs, monitoring costs and concomitant medications e.g. antiretrovirals, as is outlined in the discussion. The drivers of poor control may differ between Ugandan and South African clinics.  We added the following sentence to the discussion in response to point a:  “However, INR control was poor at South African anticoagulation services as well, despite patients not having to pay for INR testing or warfarin.”  In response to point b we have edited the sentence speaking to contribution of HIV as follows: “In addition, high HIV prevalence may further complicate warfarin dosing due to drug-drug interactions with non-nucleoside reverse transcriptase inhibitors and protease inhibitors.” |
| Study limitations. How about the sample size? Was it enough to answer the primary objective? This is not justified in the manuscript and could feature as a limitation. | This was purely an audit of current anticoagulation practice and anticoagulation control. Since no particular hypothesis was being tested, a sample size calculation is not appropriate.  We did not perform a formal sample size calculation. We aimed to review at least 200 clinical records. This target has been added to the methods |
| Conclusion: The findings from our work will inform further research to develop and validate a warfarin dosing algorithm for use in this setting. Was there any evidence to suggest that the absence of a validated algorithm affected anticoagulation control? As some sites had adopted some algorithm, one would expect the authors to have analysed if the lack of an algorithm affected anticoagulation control before having this conclusion. | None of the sites made use of a locally validated algorithm for warfarin dose selection or dose titration.  There is evidence from other settings that warfarin dosing algorithms improve anticoagulation control –see for example Pirmohamed M, Burnside G, Eriksson N, Jorgensen AL, Toh CH, Nicholson T, et al. A Randomized Trial of Genotype-Guided Dosing of Warfarin. N Engl J Med. 2013. 19;369(24):2294–303. doi: 10.1056/NEJMoa1311386 |

**Additional change to manuscript**

Our study funder requested us to update the financial disclosure statement, which now reads: “This research was commissioned by the National Institute for Health Research (NIHR) ~~[NIHR~~ Global Health Research Group on Warfarin anticoagulation in patients with cardiovascular disease in sub-Saharan Africa [ref: 16/137/101] using UK aid from the UK Government. The views expressed in this publication are those of the author(s) and not necessarily those of the NIHR or the Department of Health and Social Care. ~~The funders had no role in study design, data collection and analysis, decision to publish, or preparation of the manuscript.~~”

We have also deleted this funding disclosure statement from the manuscript text, as per journal requirements.

Sincerely,

Karen Cohen
