# Supplementary material for: A cross-sectional evaluation of five warfarin anticoagulation services in Uganda and South Africa
Source: PLoS One. 2020 Jan 29;15(1):e0227458. doi: 10.1371/journal.pone.0227458 (PMC6988943; doi:10.1371/journal.pone.0227458)
Supplement: Attachment — Submitted filename: Response to reviewers.docx [file pone.0227458.s005.docx]

09 December 2019

Joel Msafiri Francis, MD, MS, PhD

Academic Editor

PLOS ONE

**PONE-D-19-22709R1**

**A cross-sectional evaluation of five warfarin anticoagulation services in Uganda and South Africa**

Dear Dr. Francis,

Thank you for the opportunity to make further changes to our manuscript.

We respond as follows (please note that line numbers refer to the file with tracked changes):

| **Comment** | **Response** |
| --- | --- |
| ***Editor***: 1. Why no formal sample size estimation was carried out? This is critical and justification is important. | This was a descriptive audit of current service provision at different anticoagulation clinics. We did not test any hypothesis, nor did we aim to estimate any parameter to a specific level of precision. As such, there was no statistical basis on which to conduct a sample size calculation.  Instead, we considered it feasible to review at least 200 clinical records, and set this number as an aimed sample size.  We expanded the manuscript section to describe this reasoning (lines 102 to 104):  We did not perform a formal sample size calculation as this was a descriptive audit of service provision, and as we did not want to test a hypothesis or estimate a parameter with a certain level of precision. We aimed to review at least 200 clinical records as this was considered feasible. |
| ***Editor***: 2. I am not sure it is true that there was no sampling frame? Ideally – the sampling frame should have been the number of patient records available at the sites. So, the statement that there was no sampling frame may not be entirely true. | None of the clinics had a record-keeping system from which we could create a list of all patients in care at that clinic, from which to sample. Therefore, we could not draw a truly random sample of patients in care at any of the clinics.  We expanded the manuscript section to reflect this in more detail (lines 95 to 99):  The method of sampling clinic records varied between clinic sites because of difference in systems for record storage and documentation of clinic attendance. We aimed to minimise sampling bias. However, none of the included anticoagulation clinics had a readily available sampling frame that included all the patients in care at that clinic. We therefore could not draw a truly random sample of patients in care at any of the clinics. ~~We aimed to minimise bias but did not have a sampling frame from which to draw a truly random sample at any of the included clinics~~. |
| ***Reviewer #3:*** Comments have been addressed.  However, authors are advised to check the whole manuscript for consistency of their wordings. For instance, the use of "INR control" continued despite of the fact that authors agreed to replace it. Similarly, the word "interview" still appears in the abstract. | We thank the reviewer for pointing out these inconsistencies.  We made the following changes in response to this comment:   - Line 35: "We ~~interviewed~~ collected information from key staff…" - Line 119: "All ~~interviewed~~ staff providing information in Uganda…" - Line 121: "… from ~~interviewed~~ staff providing information was waived…" - Line 168: "TTR ~~INR control~~ was suboptimal at all included sites." - Line 190: "However, TTR ~~INR control~~ was poor at South African anticoagulation services as well, …" - Line 198: "… suggesting that there are other factors contributing to poor TTR ~~INR control~~."   We still use the term "INR control" in contexts where we intend the more general meaning, specifically in lines 56, 57, 66, 178, 186, and 222. |

Sincerely,

Karen Cohen
